# New type of borneol-based fluorine-free superhydrophobic antibacterial polymeric coating

**DOI:** 10.1080/15685551.2021.1924959

**Published:** 2021-05-07

**Authors:** Xin Chen, Yuexing Chen, Sengwei Lv, Lingling Zhang, Rulan Ye, Chuchu Ge, Dayun Huang, Sihai Zhang, Zaisheng Cai

**Affiliations:** a Key Laboratory of Science and Technology of EcoTextile, Donghua University, Ministry of Education, Shanghai, China; b Department of Chemistry, Lishui University, Lishui, China; c Lishui Ecological Environment Monitoring Center, Lishui Environmental Protection Bureau, Lishui, China

**Keywords:** Borneol, superhydrophobic, fluorine-free, durable, antibacterial adhesion

## Abstract

A new type of superhydrophobic borneol-based polymeric coating has been prepared. The chemical composition of the polymer particles was analyzed by Fourier transform infrared spectroscopy and X-ray photoelectron spectroscopy, which showed that the polymer did not contain fluorine, which can effectively avoid the cytotoxic risk of fluorine. By dynamic light scattering, scanning electron microscopy, and static contact angle measurement, the contact angle of the prepared coating gradually increased with increasing diameter of the polymer particles, and a superhydrophobic coating surface was finally obtained. Interestingly, after dissolving the superhydrophobic sample with tetrahydrofuran and making it a normal hydrophobic sample, the antiadhesion performance for *E. coli* was greatly reduced, and it could not effectively prevent *E. coli* adhesion. In addition, a long-term antiadhesion study of bacteria was performed. The superhydrophobic borneol-based polymer coating showed long-term resistance to *E. coli* adhesion. Therefore, the excellent antibacterial properties and cell compatibility mean that this series of polymer materials has great potential in the field of biomedicine.

## 1. Introduction

Contamination of medical equipment by bacteria is one of the reasons for infections in hospitals, and adhesion of bacteria is the first step in bacterial colonization or proliferation, and also in bacterial biofilm formation. Therefore, prevention of bacterial adhesion to the surface of materials is an important research topic [1]. There are two types of antibacterial coatings: antibacterial coatings and bactericidal coatings. Antibacterial coatings are usually based on nonpolluting polymers, such as polyethylene glycol and its derivatives [2], and zwitterionic polymers [3]. Bactericidal coatings are based on surface-attached biological bactericides, such as antimicrobial peptides [4], or preloaded or embedded biological insecticides, such as quaternary ammonium compounds [5] and metal nanoparticles [6], to kill bacteria attached to the surface or suspended near the surface, providing a direct method to prevent bacteria from attaching. However, research is mainly focused on immobilization or release of bactericidal substances [7–11], and these materials are still not satisfactory because of their limited efficiency, cytotoxicity, and antibiotic resistance [12]. Moreover, metal nanoparticles are toxic to bacteria and eukaryotic cells, such as human tissue cells. For example, silver is only nontoxic to cells at a very small concentration, and the cytotoxicity of silver nanoparticles increases with increasing concentration [13]. In addition, weak interactions such as hydrogen, van der Waals causes aggregate phenomena of metal nanoparticles to occur [14].

From the perspective of the internal mechanism, most existing antibacterial materials mainly achieve antibacterial effects by interacting with phospholipid components to destroy the integrity of cell membranes or affect the function of bacteria [8,15,16]. However, the use of the ‘feel’ of bacterial cells on the surface of the material to induce subsequent selective attachment as a new type of biomedical material and interface design method has rarely been reported. Studies have shown that the interface of chiral materials has a great influence on cell adhesion and protein adsorption [17]. When cells are attached to the outer surface, they have a clear tendency to have different chiral stereochemistry [18]. In recent years, the natural compound borneol has become an ideal antibacterial adhesion material because of its unique chiral characteristics, excellent antibacterial adhesion, and various biomedical functions [1,19]. Shi et al. [20] synthesized a borneol-grafted cellulose material by covalently grafting borneol on the surface of cellulose. The polymer showed excellent antifungal adhesion and fungal growth inhibition performance. It was also safe, and had the advantages of easy preparation and good biocompatibility. Wu et al. [21] used the thiol-ene Michael addition reaction to prepare a polyurethane coating material containing dihydroxy-terminated isobornyl acrylate, and they then performed antibacterial adhesion experiments by plate counting and optical density (OD) measurements. The results showed that the coating effectively resisted bacterial adhesion. Its antiadhesion effects on *Escherichia coli* and *Staphylococcus aureus* were 89.3% and 80.4%, respectively. Although these coated surfaces can prevent or reduce initial adhesion of bacteria, they cannot achieve 100% prevention of bacterial adhesion. Once bacteria adhere to a surface, colonies will inevitably form, and a biofilm will finally form.

Superhydrophobic surfaces have recently attracted widespread attention in wastewater treatment and biomedical applications because of their antipollution, antiadhesion, self-cleaning, and anticorrosion properties [22–26]. A superhydrophobic surface refers to a surface where the static contact angle of water is greater than 150°. Owing to its high hydrophobicity, a superhydrophobic surface has advantages in resisting protein, bacteria, and cell adhesion [27,28], and it can be used as an antiadhesion coating. For example, Privett et al. [29] prepared a superhydrophobic xerogel coating using a mixture of nanostructured silica colloid, fluoroalkoxysilane, and a silane matrix that can reduce adhesion of bacteria by about two orders of magnitude compared with normal hydrophobic surfaces. Therefore, compared with normal hydrophobic surfaces, superhydrophobic surfaces can effectively resist bacterial adhesion, thereby reducing the initial bacterial adhesion and inhibiting formation of a biofilm. However, superhydrophobic materials generally contain fluorine components, which have certain cytotoxicity and cannot be degraded in the body [30]. Ideally, an antibacterial surface should be superhydrophobic and able to prevent adhesion of bacteria in the absence of antibiotics, and have good biocompatibility and biodegradability.

In this study, a new type of polymer coating was prepared using borneol as the substrate. The surface-structure characteristics and thermal stability of the coating material were characterized by Fourier transform infrared (FTIR) spectroscopy, scanning electron microscopy (SEM), and thermogravimetric analysis (TGA), and the static contact angle of the polymer coating surface was measured. In addition, through antibacterial adhesion experiments, the antibacterial adhesion performance of the coating material was investigated in detail. The coating material prepared in this study is superhydrophobic, but it does not contain fluorine, which avoids the cytotoxic risk of fluorine. The coating material also combines the antibacterial and superhydrophobic properties of borneol, shows unique antibacterial adhesion properties, and can effectively prevent bacterial adhesion and biofilm formation.

## 2. Materials and methods

### 2.1. Materials

Sodium dodecyl sulfate (SDS), isobornyl acrylate and isomeric hexadecane (HD) were purchased from Shanghai Maclean Biochemical Technology Co. All of the aqueous solutions were prepared using laboratory-prepared deionized water (0.055 μΩ). L929 mouse fibroblasts were obtained from Wenzhou Longbo Biotechnology Co.

### 2.2. Preparation of the polymer (Fig S1)

In the first step, SDS (0.25 g), HD (0.835 g), and water (85 mL) were added to a 250 mL three-necked flask. The mixture solution A was then stirred in an oil bath at 70 °C for 30 min while passing N_2_ and then introduced into a condenser. Then the mixture solution A was cooled to room temperature and 13 g isobornyl acrylate (BA) was added into the flask. After ultrasonic treatment for 3 min at 0 °C, the KPS aqueous solution (0.05 g of KPS in 5 ml distilled water) was added, and the polymerization was allowed to proceed for 8 h at 70 °C to obtain to produce seed latex.

In the second step, water (20 mL), SDS (0.35 g), and prescribed amount of isobornyl acrylate (see Table 1) were introduced into a 50 ml three-neck round-bottom flask equipped with a mechanical stirrer, dropping funnels and a nitrogen inlet and stirred for 30 min to produce mixture solution B. The obtained mixture solution B and the KPS solution (0.003 g/mL) were added continuously into the reactor containing seed latex over a period of 3 h at 80 °C. After polymerization for 30 min, the solids were collected and washed with water and ethanol. Then the polymer particles were re-dispersed in ethanol to obtain a latex with a solid.

**Table 1.** Sample compositions

Samples	the First Step (g)			the Second Step (g)		Further
	SDS	HD	BA	SDS	BA	processing
**PLB-1**	**0.25**	**0.835**	**13**	**0.35**	**4**	**none**
**PLB-2**	**0.25**	**0.835**	**13**	**0.35**	**8**	**none**
**PLB-3**	**0.25**	**0.835**	**13**	**0.35**	**12**	**none**
**PLB-4**	**0.25**	**0.835**	**13**	**0.35**	**12**	a

a After preparation of the PLB-3 film, the film was dissolved with tetrahydrofuran to form the PLB-4 film.

### 2.3. Preparation of the coatings

The obtained polymer nanoparticles were separated by centrifugation and dispersed in water to obtain a certain concentration of polymer nanoparticles. First, a layer of adhesive was uniformly spin-coated on a clean slide, and then the polymer nanoparticles were directly cast on the adhesive to prepare polymer nanoparticle films. Finally, the films were dried at room temperature of 25 °C and relative humidity of 40%, and then transferred to a vacuum drying oven for another 24 h.

### 2.4. Characterization

The FTIR spectra of the samples were recorded with an EQUINOX55 FTIR spectrometer (Bruker, Rheinstetten, Germany). XPS analysis of the thin-film surfaces was performed with a Perkin Elmer ESCA 5600 spectrometer (Perkin Elmer, Shelton, CT, USA) using a Mg Kα x-ray source (1253.6 eV) from 0–1200 eV with a take-off angle of 0°. The nuclear magnetic resonance (NMR) measurements of the polymer were carried out with a Varian Inova 400 NMR spectrometer. In order to obtain purified samples, the samples were demulsified and precipitated with 20% CaCl_2_ solution. The extracted copolymers were washed with water and ethanol, and then dissolved in tetrahydrofuran. After that, the copolymers were settled out with methanol by sedimentation and then dried. The gel permeation chromatography (GPC) was carried out of Waters 2695. The dynamic light scattering (DLS) measurements of the polymer particles were carried out with a Malvern Zetasizer Nano-ZS90 instrument at room temperature. A scanning electron microscope (JEOL JSM-7800 F) was used to observe the surface morphologies of the thin-film samples. TGA of the samples was performed with a NETZSCH TG 209 thermal analyzer. A differential scanning calorimeter (Shimadzu DSC-60A, Japan) was used for thermal analysis of the samples.

The *E. coli* strain was placed in 50 mL of sterile liquid medium and incubated overnight at 37 °C with shaking at 200 rpm. Finally, the *E. coli* solution was diluted to a concentration of 10^6^ CFU/mL with sterile phosphate-buffered saline (PBS).

The ability of the film samples to resist *E. coli* adhesion was evaluated. A sterile poly(methyl methacrylate) (PMMA) sheet was placed at an angle to the film sample and immersed in 10^6^ CFU/mL of the diluted bacterial solution for 4 h at a constant temperature of 37 °C. The sample was then removed and gently rinsed 10 times with PBS solution to remove any unadhered bacteria. The sample was then placed in a new PBS solution tube and sonicated at a constant temperature of 37 °C for 5 min to remove the bacteria from the surface of the sample. The tube was shaken to mix the bacteria in the PBS solution, and then the solution was removed and serially diluted and spread on agar plates. After incubation for 24 h, the numbers of bacteria on the agar plates were counted by plate counting.

The OD was used to study the above-mentioned bacterial attachment. Five groups of samples (three sterile PMMA sheets, and PLB-1, PBL-2, PBL-3, and PBL-4 film samples) were placed at an angle and immersed in 10^6^ CFU/mL of dilute bacterial solution in a 37 °C thermostat. A set of samples was taken every 3 h after three gentle rinses with PBS and then incubated at 37 °C for 4 h in fresh liquid medium. The OD of a 200 µL sample was then determined at a measurement wavelength of 600 nm to measure the change in the OD value. In total, bacterial adhesion on the samples was measured for 24 h.

The in vitro cytotoxicity–indirect cytotoxicity assay was used to determine the membrane cytotoxicity. L929 mouse fibroblasts (ATCC NCTC clone 929:CCL 1) were cultured with 10% fetal bovine serum in Dulbecco’s modified Eagle’s medium. The culture conditions were 5% CO_2_, 37 °C, and relative humidity above 90%, and the membranes were immersed in the medium for 72 h. The membranes were then interacted with L929 cells at a density of 1 × 10^5^ cells/mL in 96-well plates overnight. After 24 h, the medium of the membranes was changed to 10% 3-(4,5-dimethylthiazol-2-yl)-2,5-diphenyltetrazolium bromide reagent (MTT) and they were incubated at 37 °C for 4 h. The membranes were then incubated with L929 cells at a density of 1 × 10^5^ cells/mL in 96-well plates. The cultures were removed and incubated with 100 µL propylene glycol solution (0.04 M HCl) at 37 °C for 4 h. The cell viability was assayed by MTT reduction and the absorbance was measured at 570 nm using a microplate reader.

## 3. Results and discussion

**Figure 1.**
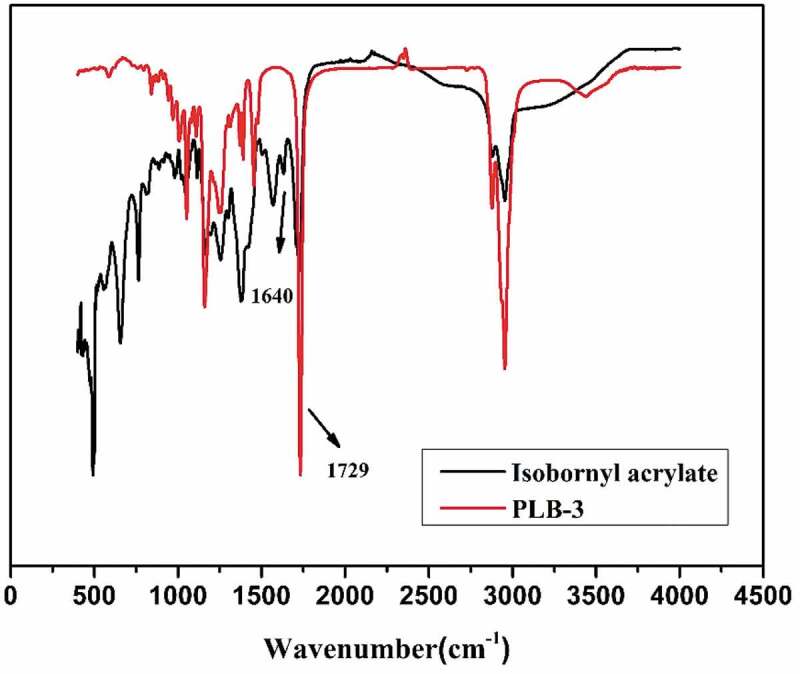
FTIR spectra of isobornyl acrylate and PLB-3

FTIR spectroscopy was performed to characterize the functional groups of the monomer and polymers and their chemical components. Because PLB-1, PLB-2, and PLB-3 had the same composition, PLB-3 was selected as a representative sample for the FTIR and XPS measurements. The FTIR spectra of monomeric isobornyl acrylate and the borneol-based PLB-3 polymer are shown in Figure 1 (see Table 1 for the sample compositions). In the FTIR spectra of both monomeric isobornyl acrylate and PLB-3, there are vibrational peaks at 1729 cm^−1^, which are caused by the C = O stretching vibration of the isobornyl acrylate ester group. For the isobornyl acrylate monomer, there is a distinct vibrational peak at 1640 cm^−1^ (the stretching vibration peak of the C = C bond), which is not present in the FTIR spectrum of the PLB-3 polymer. This confirmed that the double bond of isobornyl acrylate reacted.

**Figure 2.**
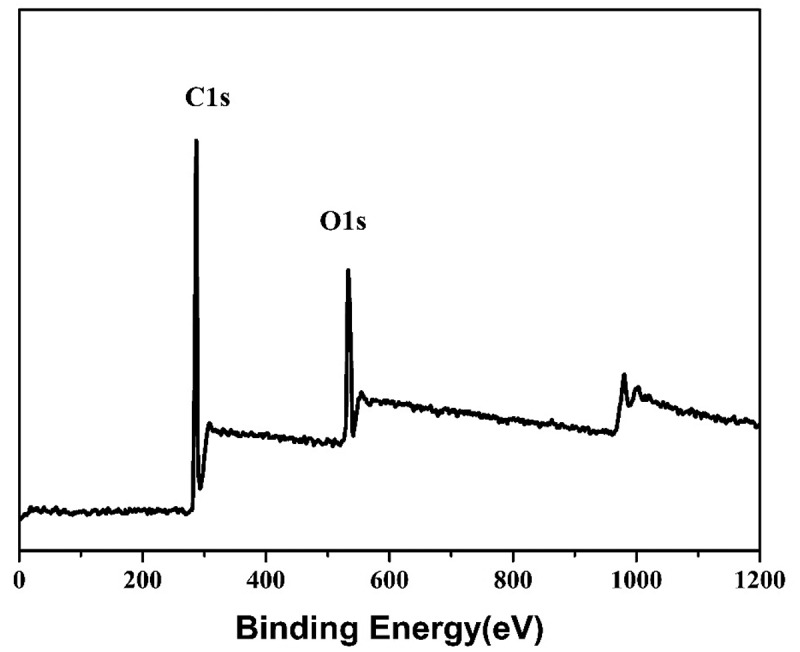
XPS spectrum of PLB-3

XPS of PLB-3 was performed, and the XPS spectrum is shown in Figure 2. There are two clear peaks in the spectrum, namely, C 1s (283.9 eV) and O 1s (532.9 eV). There is no peak at 689.2 eV, indicating the absence of F-containing elements in the polymer. It has been shown that long-term exposure to F and its compounds can damage the brain, bones, kidneys, teeth, and other organs [31]. For example, as a typical POP, perfluorooctane sulfonic acid is persistent, bioaccumulative, and toxic to mammalian species [32]. Therefore, the fluorine-free polymeric nanoparticles prepared in this study can effectively avoid the cytotoxicity of elemental F and are more environmentally friendly than F-containing polymers.

**Figure 3.**
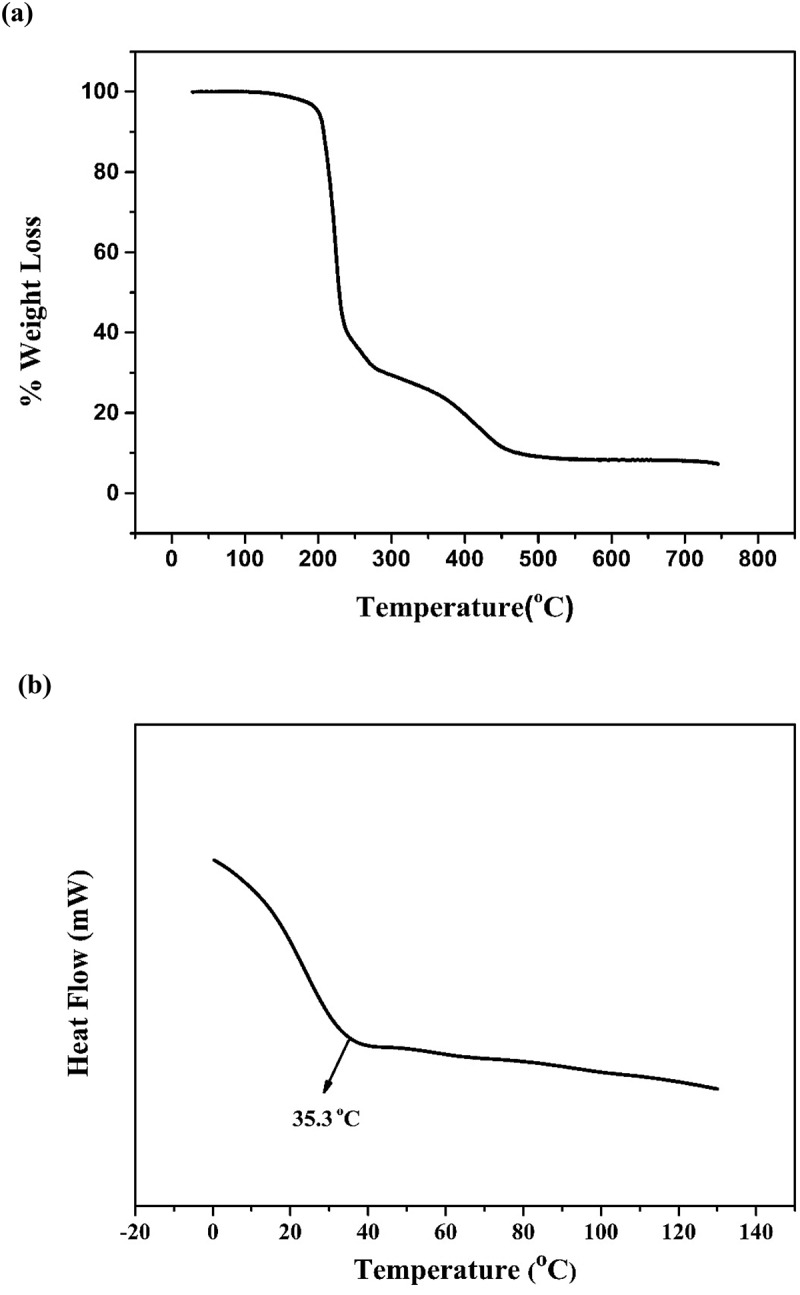
(a) TGA and (b) DSC curves of PLB-3

The thermal stability of PBL-3 was investigated by TGA, and the weight loss curve is shown in Figure 3(a). The curve showed two thermal degradation stages. The first stage was cleavage of the pendant groups of the polymer fragments in the temperature range 190–280 °C. The second stage was decomposition of the polymer backbone in the range 280–520 °C. In the thermal degradation curve of PBL-3, the temperature before degradation of PBL-3 reached 5 wt% was 201 °C. The thermal properties of PBL-3 were investigated by DSC, and the results are shown in Figure 3(b). The glass-transition temperature (*T*_g_) of PBL-3 was 35.3 °C.

Compared with the HNMR spectra of the monomers (A cited in reference 1), the polymer spectra have no corresponding peaks of monomers (a and b), indicating that the double bond of the polymer has been completely reacted (Figure S4). 7.26 is the characteristic peak of deuterium chloroform, not the H characteristic peak of olefin. What’s more, reference to the polymer chemical equation, the other H can be marked in the NMR spectra, indicating the successful preparation of the polymer. Using gel permeation chromatography (GPC, Waters 2695), the average molecular weights of the polymers were found to be approximately Mn = 92,268 g/mol, Mw = 327,691 g/mol, Polydispersity = 3.55(Figure S5).

**Figure 4.**
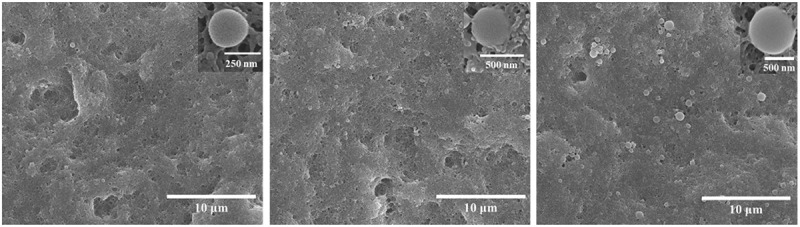
SEM images of PLB-1, PLB-2, and PLB-3

**Figure 5.**
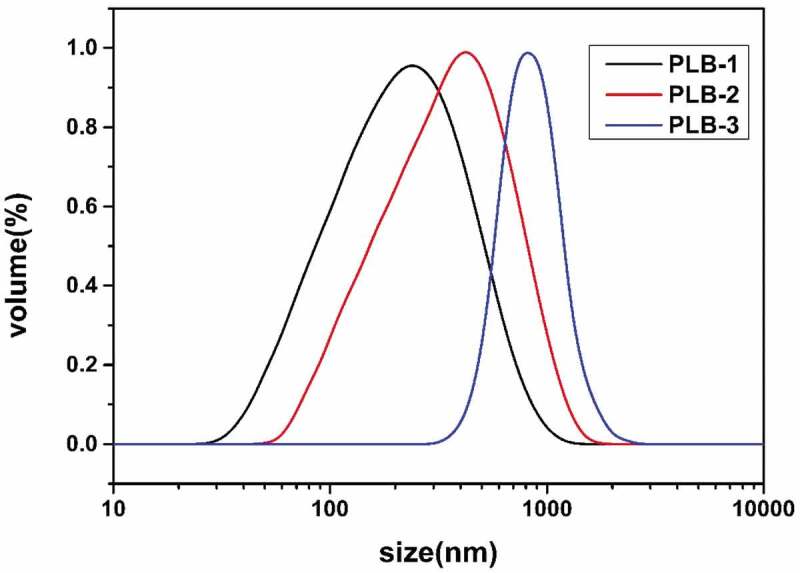
DLS curves of PLB-1, PLB-2, and PLB-3

The surface morphologies of the PLB-1, PLB-2, and PLB-3 film samples were characterized by SEM, and the results are shown in Figure 4. The surface structures of the three film samples were dense and relatively rough. Magnification of the film samples (see the inserts of Figure 4) showed that PLB-1, PLB-2, and PLB-3 were spherical in shape, and the radius of the polymer microspheres gradually increased with increasing isobornyl acrylate content. The DLS curves (Figure 5) showed the same results, and the particle sizes of the synthesized PLB-1, PLB-2, and PLB-3 polymer particles were 249, 432, and 759 nm, respectively. Thus, the particle size of the polymer gradually increased with increasing isobornyl acrylate content.

**Figure 6.**
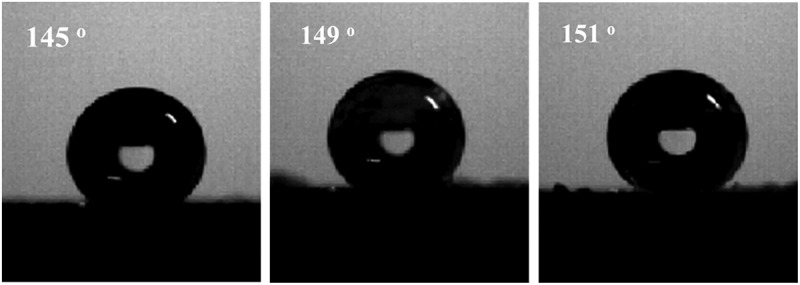
Contact angles of PLB-1, PLB-2, and PLB-3

**Figure 7.**
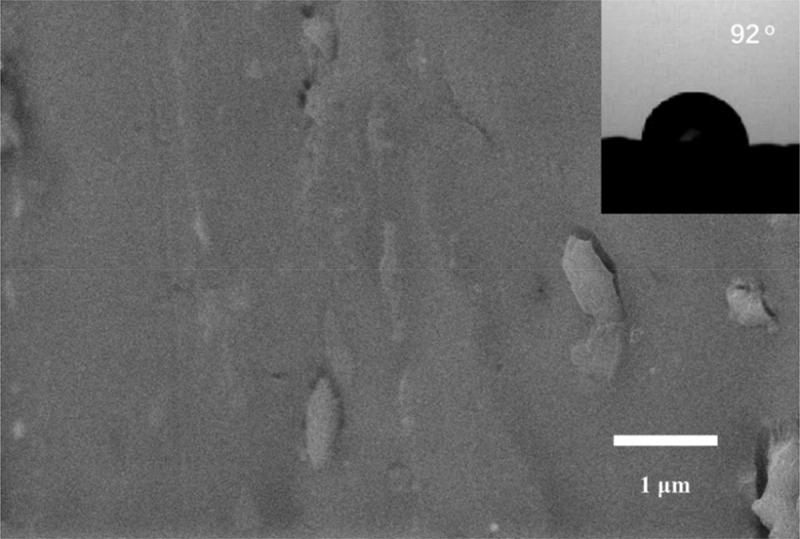
SEM image and contact angle of PLB-4

Static contact angle measurement is a method to evaluate the hydrophobic properties of a material surface, and the static contact angle of the film surface increases as the wetting resistance increases. The static contact angles of the three polymer films were measured, and the results are shown in Figure 6. The static contact angles of the surfaces of the PLB-1, PLB-2, and PLB-3 film samples were 145°, 149°, and 151°, respectively. This is because with increasing polymer nanoparticle size, the coating surface morphology changes from a nanostructure to a multi-scale micro/nanostructure, which affects the hydrophobicity of the coating surface to some extent. To further investigate the effect of the polymer-film surface structure on the magnitude of the static contact angle, the surface of the PLB-3 film sample was reconstituted into a thin film (PLB-4) by dissolving it with tetrahydrofuran. A SEM image of the PLB-4 thin-film sample and the measured static contact angle are shown in Figure 7. No spherical polymer was observed on the surface of the film, and the static contact angle was 92°, indicating a change from a superhydrophobic surface for PLB-3 to a normal hydrophobic surface for PLB-4.

**Figure 8.**
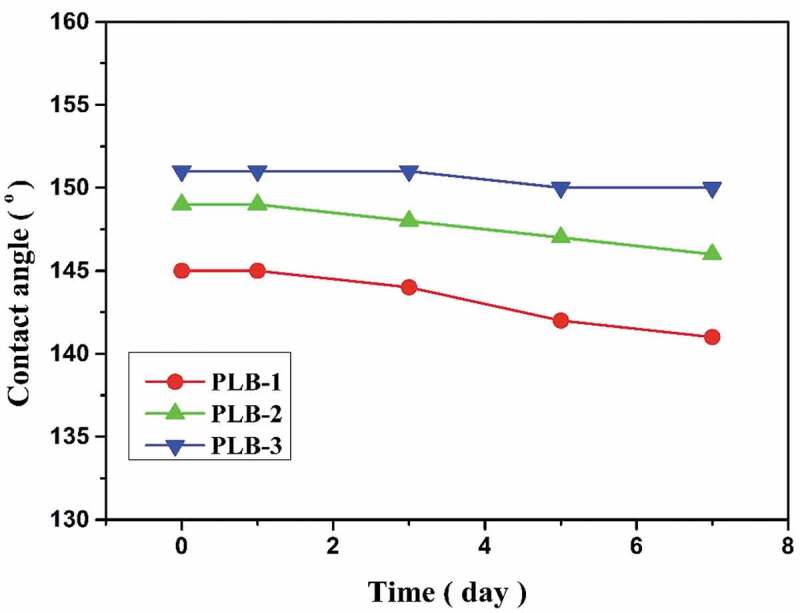
Relationships between the static contact angle and the immersion time for PLB-1, PLB-2, and PLB-3

To further investigate the effect of prolonged immersion in water on the hydrophobic properties of the polymer-film surfaces, the changes of the contact angles of the polymer films at room temperature (25 °C) with the water immersion time were determined (Figure 8). At the beginning, the static contact angles of the surfaces of the PLB-1, PLB-2, and PLB-3 film samples were 145°, 149°, and 151°, respectively. The contact angles of the three samples slightly decreased with increasing immersion time. After 7 days of immersion in water, the static contact angles of the PLB-1, PLB-2, and PLB-3 film surfaces were 141°, 146°, and 150°, respectively. From the experimental results, all three films showed excellent stability, and the PLB-3 film showed the best stability. The static contact angle of the PLB-3 film surface was 150° after 7 days of water immersion, indicating that the superhydrophobic property of the surface was maintained.

**Figure 9.**
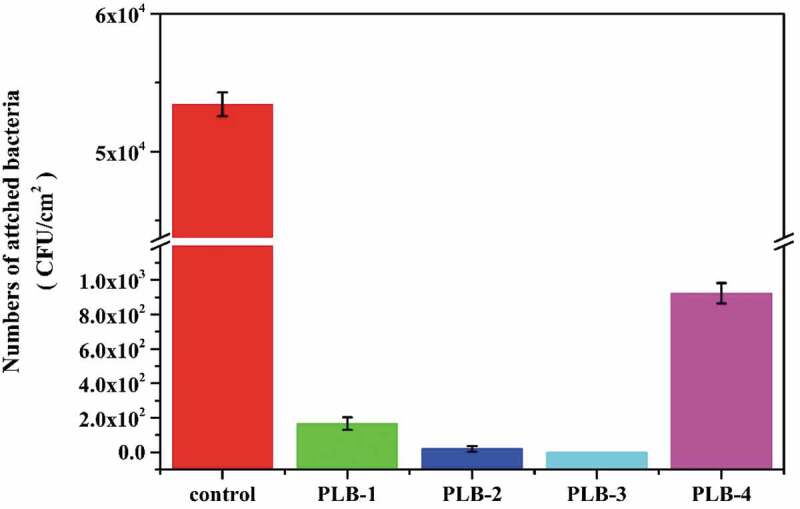
Antiadhesion test results

The antibacterial adhesion properties of the polymer-film samples were evaluated in the presence of *E. coli*, and the bacterial plate counts are shown in Figure 9. The number of *E. coli* adhering to the surface of the control sample was 5.3 × 10^4^ CFU/cm^2^, while the numbers of bacteria adhering to the surfaces of the PLB-1, PLB-2, PLB-3, and PLB-4 polymer samples were 167, 20, <10, and 923 CFU/cm^2^, respectively. These results clearly indicate that the polymer films containing the borneol component showed good resistance to bacterial adhesion. In addition, the superhydrophobic surface of the PLB-3 sample was able to more effectively inhibit bacterial adhesion on the surface compared with the hydrophobic PLB-4 sample. From the plate count results, the number of bacteria adhering to the surface of the PLB-3 sample was more than two orders of magnitude lower than that of the PLB-4 sample. Moreover, by comparing the PLB-1 and PLB-2 samples, the antibacterial adhesion effect of the film surface was better with increasing static contact angle of the surface.

**Figure 10.**
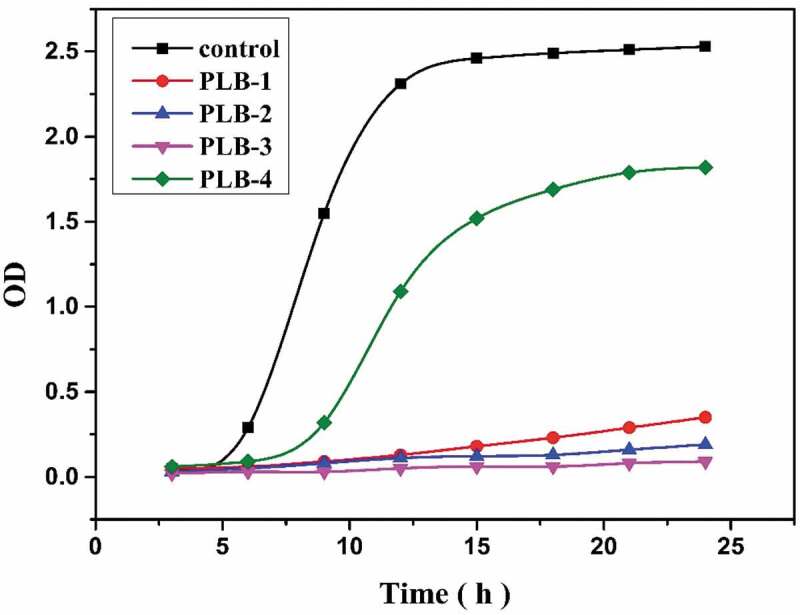
Optical density (OD 600) test results of the samples

The OD test results of *E. coli* adhered to the polymer-film samples after further incubation are shown in Figure 10. After incubation for 12 h, the control liquid medium showed a cloudy bacterial suspension, PLB-4 was translucent, and PLB-1, PLB-2 and PLB-3 were very clear in appearance. The corresponding OD values showed that the bacterial density was in the order (from low to high) control, PLB-4, PLB-1, PLB-2, and PLB-3. In summary, both antibacterial adhesion experiments confirmed that the polymers containing isobornyl acrylate groups showed good resistance to microbial adhesion, and the antibacterial adhesion performance of the polymer film was better for higher isobornyl-group content. The internal mechanism of antimicrobial adhesion may be due to the ‘chiral taste’ characteristic of microorganisms, and therefore the chiral biological interface has a great influence on bacterial adhesion. Moreover, the film samples prepared in this study also combined the antiadhesion effect of the superhydrophobic surface, making it very difficult for bacteria to adhere to the surfaces of the films and the films being very effective in preventing formation of bacterial biofilms on their surfaces. The films were also superior to PLB-4 with a normal hydrophobic surface.

**Figure 11.**
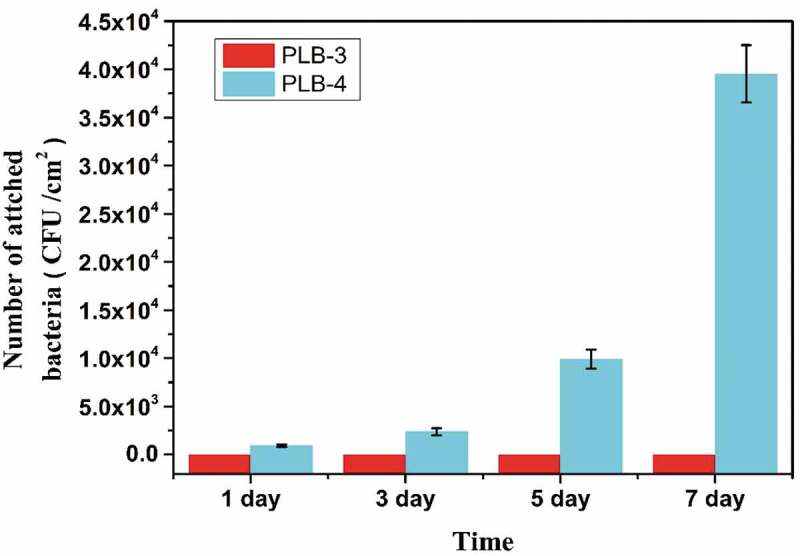
Long-term antibacterial adhesion of *E. coli* to PLB-3 and PLB-4

To investigate the long-term antiadhesion ability of the polymer films against bacteria, 12 groups of PLB-3 and PLB-4 film samples were placed in test tubes containing 10 mL of 5 × 10^3^ CFU/mL *E. coli* suspension, trypticase soy broth, and glucose in PBS. After a period of incubation in a constant temperature incubator at 37 °C, three groups of samples were removed and the numbers of surface bacteria adhering to the samples were counted by the plate-counting method. The results are shown in Figure 11. The superhydrophobic PLB-3 film sample still showed a good antibacterial adhesion effect after 7 days, while the hydrophobic PLB-4 film sample could not completely prevent bacterial adhesion on the first day, and showed exponential bacterial growth with time. The experimental results indicate that the superhydrophobic PLB-3 film sample showed a long-term antibacterial adhesion effect.

**Figure 12.**
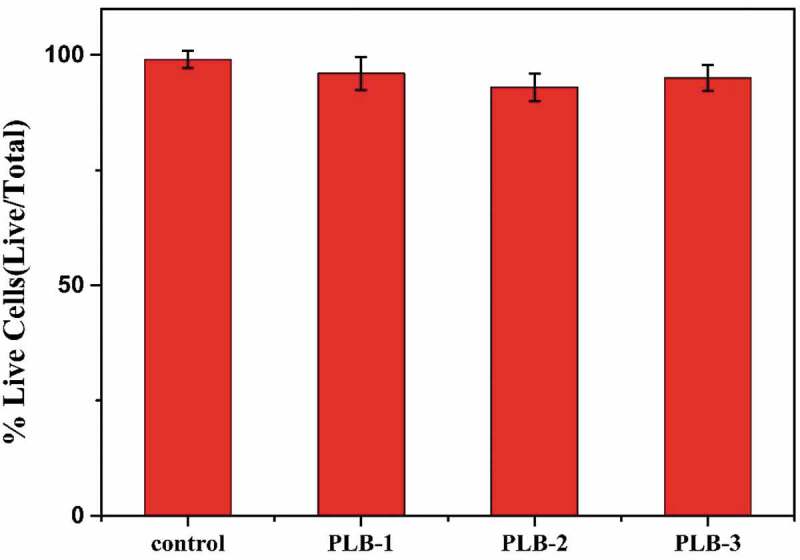
MTT results of PLB-1, PLB-2, and PLB-3

To investigate the cytotoxicity of the film samples, MTT-based analysis was performed, and the results are shown in Figure 12. Using PMMA as the control group, the cell survival rate of PMMA was close to 100%, while the cell survival rates of the PLB-1, PLB-2, and PLB-3 polymers were 96%, 93%, and 95%, respectively, indicating good cytocompatibility. Therefore, the superhydrophobic polymeric film materials prepared in this study, which do not contain fluorine, have good biocompatibility and resistance to bacterial adhesion, and they have promising applications in the biomedical field.

## 4. Conclusion

We have developed novel borneol-based polymers as antimicrobial materials or coatings. The components of the polymers were analyzed by FTIR spectroscopy and XPS. The results showed that the polymers did not contain F, which can effectively avoid the cytotoxicity of F and means that the polymers are more green than F-containing polymers. TGA and DCS showed that the polymer materials had excellent thermal stability. The static contact angle showed that the PLB-3 film sample possessed superhydrophobic properties. An antiadhesion test using *E. coli* showed that the PLB-3 film sample had excellent antiadhesion performance against bacteria. The PLB-3 thin-film sample was dissolved with tetrahydrofuran to prepare a normal hydrophobic thin-film sample (PLB-4). The hydrophobic PLB-4 film was not completely resistant to *E. coli* adhesion, and bacteria gradually adhered to the surface with time, while the PLB-3 superhydrophobic film maintained stable long-term antiadhesion performance. More importantly, the polymers also showed good cytocompatibility. Therefore, the antimicrobial films prepared in this study will promote research and application of antimicrobial materials in the biomedical field.

## Data Availability

The raw/processed data required to reproduce these findings can’t be shared at this time as the data also forms part of an ongoing study.
